# Prädiktoren von prozessbezogener und strukturierender elterlicher Unterstützung während des (coronabedingten) Distanzunterrichts

**DOI:** 10.1007/s11618-021-01015-6

**Published:** 2021-04-15

**Authors:** Andreas Sander, Laura Schäfer, Stefanie van Ophuysen

**Affiliations:** Institut für Erziehungswissenschaft, Arbeitsgruppe Forschungsmethoden/empirische Bildungsforschung, Westfälische-Wilhelms-Universität Münster, Georgskommende 33, 48143 Münster, Deutschland

**Keywords:** COVID-19, Schulschließung, Familiäre Lernbegleitung, Strukturierende/prozessbezogene Unterstützung, Lehrkrafthandeln, COVID-19, Family learning support, School closure, Structuring/process-related support, (Perceived) Teacher involvement

## Abstract

Die Schulschließungen aufgrund der Corona-Pandemie verlagerten das schulische Lernen in die Familien. Schüler*innen standen vor der Aufgabe, ihr Lernen und ihre Lernzeit eigenständiger zu gestalten und wurden dabei vornehmlich von ihren Eltern sowohl organisatorisch als auch fachlich begleitet. Auf Basis bisheriger Forschung zur elterlichen Hausaufgabenunterstützung und zu Bildungspartnerschaften ist anzunehmen, dass sowohl die strukturierende als auch die prozessbezogene Lernbegleitung durch die Eltern von soziodemographischen Merkmalen der Familie abhängen, aber ebenfalls mit Merkmalen des Kindes (z. B. Alter, Lernverhalten) und der wahrgenommenen schulischen Lernbegleitung (Engagement der Lehrkraft, Anforderungsniveau) assoziiert sind. Diese postulierten Zusammenhänge wurden auf Basis von Daten einer standardisierten Onlinebefragung mit *N* = 6685 Eltern von Kindern an weiterführenden Schulen in Nordrhein-Westfalen regressionsanalytisch untersucht. Die Befunde sprechen für eine adaptive Lernbegleitung der Eltern, die für ältere, weibliche Kinder mit günstigem Lernverhalten weniger intensiv ausfällt als für jüngere, männliche Kinder mit ungünstigem Lernverhalten. Auch die Lernbegleitung durch die Schule trägt zur Vorhersage des elterlichen Verhaltens bei, wobei engagiertes Lehrkraftverhalten gerade bei Familien mit nicht-gymnasialer Schulbildung der Eltern zu verstärkter Unterstützung des Kindes beiträgt.

## Abstract (en)

School closure due to Corona-pandemic shifted school learning into the families. Pupils were faced with the task to organize learning and the use of learning time more independently. The children were primarily supported by parents in aspects of organization and learning content. Based on previous research on parental homework support and educational partnerships, it can be assumed that both the structuring and process-related learning support provided by parents depends on socio-demographic characteristics of the family, but is also associated with characteristics of the child (e.g., age, learning behavior) and the perceived school learning support (teacher commitment, level of requirements). These associations were investigated by regression analysis on the basis of data from a standardized online survey with *N* = 6685 parents of children at secondary schools in the German federal state of North Rhine-Westphalia. The findings speak in favor of adaptive learning support for parents, which is less intensive for older and female students with favorable learning behavior than for younger and male students with unfavorable learning behavior. School-based learning guidance also contributes to the prediction of parental behavior, with committed teacher behavior contributing to increased support for the child, especially in families with lower school leaving certificates of (surveyed) parents.

## Einleitung

Durch die Corona-Pandemie wurden auf staatlicher Seite einschneidende Maßnahmen ergriffen, die im sogenannten „Shutdown“ mündeten. Dieser umfasste auch die temporäre Schließung aller Schulen. Der Wegfall der Schule als Arbeits- und Lebensort von Schüler*innen verlagerte deren Lernen für ca. 6 Wochen (18.03. bis 04.05.2020 in Nordrhein-Westfalen [NRW]) vollständig in den häuslichen Bereich.

Mit Blick auf die Qualität des Lernens auf Distanz berichten Voss und Wittwer (2020), dass die kognitive Aktivierung vielfach gering ausgeprägt war, da Material – vornehmlich Arbeitsblätter – häufig in repetitiver, wenig fokussierter Weise und oft auch ohne explizite Rückmeldung zur Bearbeitungsqualität eingesetzt wurde. Ein synchroner Online-Unterricht, der mehr Möglichkeiten der kognitiven Aktivierung und der direkten Rückmeldung bietet, fand – nicht zuletzt aufgrund mangelnder technischer Ausstattung der Schulen und fehlenden Konzepten für das digitale Lernen (z. B. Eickelmann et al. 2020) – vergleichsweise selten statt. Ebenfalls wurden eher wenige Hinweise zur inhaltlichen oder zeitlichen Strukturierung des Lernens gegeben. Eltern standen entsprechend vor der Frage, wie intensiv und in welcher Form sie das Lernen ihrer Kinder begleiten und unterstützen wollen respektive können.

Diese elterliche Lernbegleitung im Kontext der Schulschließung weist vermutlich hohe Ähnlichkeiten zur Betreuung der Hausaufgaben auf, wenngleich durchaus situative Unterschiede zu berücksichtigen sind. Den Hausaufgaben werden verschiedene Funktionen zugeschrieben: Schulischer Unterrichtsstoff soll wiederholt, eingeübt und gefestigt und damit letztlich die Schulleistung verbessert werden (Mischo und Haag 2006). Lag die empfohlene Hausaufgabendauer vor den Schulschließungen in NRW bei ca. 60–75 min pro Tag (Empfehlung für die Sekundarstufe I; Bereinigte Amtliche Sammlung der Schulvorschriften NRW [BASS] 12 – 63 Nr. 3, 2015), so musste nun der Umfang von etwa 30 Schulstunden pro Woche (Stundentafel der Sekundarstufe I an Gymnasien, vgl. Ministerium für Schule und Bildung [MSB] NRW 2021) in den Familien strukturiert bzw. kompensiert werden. Bei dem häuslichen Lernen und Arbeiten während der Schulschließung stand weiterhin auch die Erarbeitung neuer Inhalte auf dem Programm, sodass Eltern sich eventuell stärker als bei den Hausaufgaben aufgerufen fühlten, diesen umfänglicheren und komplexeren Prozess zu begleiten.

Im Kontext der Forschung zur elterlichen Hausaufgabenunterstützung kristallisiert sich eine Trennung zwischen strukturierenden und prozessbegleitenden Strategien heraus. Die *strukturierende Lernbegleitung* ist durch die Organisation des Arbeitsumfeldes und die Sicherung günstiger Arbeitsbedingungen gekennzeichnet. Zu diesem strukturgebenden Verhalten der Eltern zählen Dumont et al. (2014) zum einen das Festlegen von Regeln (z. B., dass die Hausaufgaben erledigt sein müssen, bevor sich das Kind mit Freund*innen verabredet), zum anderen aber auch die Bereitstellung eines Schreibtisches und Materials sowie eines ruhigen Umfelds zum Arbeiten. Dieser Schaffung eines günstigen Arbeitsumfeldes kam in der Zeit der Schulschließung vermutlich besonderes Gewicht zu, da der gesamte Lernprozess zuhause zu organisieren war. Erschwerend kam hinzu, dass in der Regel mehrere Personen in einer Wohnung arbeiten, lernen oder spielen und sich ggf. bei der Nutzung von Räumlichkeiten oder technischer Ausstattung abstimmen mussten.

Die *prozessbezogene Lernbegleitung* bezieht sich auf die Betreuung der Kinder bei der Bearbeitung von Aufgaben. Hier ist zwischen kontrollierenden, bevormundenden Strategien einerseits und bedarfsorientierten, Unterstützung anbietenden Strategien andererseits zu unterscheiden. So kann eine kontrollierende Vorgehensweise, in der sich Eltern ungefragt und bevormundend in die Bearbeitung der Aufgaben „einmischen“, als dysfunktional gelten. Als motivational förderlich werden hingegen die strukturierende und die bedarfsorientierte Begleitung herausgestellt (vgl. Dumont et al. 2014; Moroni et al. 2015).

Befunde der Hausaufgabenforschung weisen z. T. einen negativen Zusammenhang zwischen der elterlichen Hausaufgabenhilfe und der Leistung auf (vgl. Xu et al. 2010). Dabei ist jedoch nicht die für die Hausaufgaben aufgewendete Zeit entscheidend (vgl. Trautwein et al. 2006), sondern die Qualität der elterlichen Unterstützung. So geht eine Lernbegleitung, die von Kontrolle und einer Einmischung in die Autonomie des Kindes geprägt ist, mit einer negativen Leistungsentwicklung des Kindes einher (vgl. Cooper et al. 2000). Eine autonomiefördernde und unterstützende Hausaufgabenbegleitung steht hingegen in einem positiven Zusammengang mit der Leistung (vgl. Moroni et al. 2015).

Es ist somit davon auszugehen, dass die Art der elterlichen Lernbegleitung durchaus prädiktiv für den Lernerfolg der Kinder ist. Doch von welchen Merkmalen hängt es ab, wie die Eltern strukturierend und/oder prozessbegleitend das Lernen ihrer Kinder in der Zeit der coronabedingten Schulschließung unterstützen? Dieser Frage gehen wir mit der vorliegenden Studie nach.

## Prädiktoren elterlicher Lernbegleitung

Um Annahmen über mögliche Prädiktoren der elterlichen Lernbegleitung während der coronabedingten Schulschließung in NRW abzuleiten, fokussieren wir insbesondere die Forschung zur elterlichen Hausaufgabenbetreuung sowie zur Zusammenarbeit von Schule und Elternhaus. Dabei schauen wir zunächst auf soziodemographische Merkmale der Familie. Doch ebenso, wie sich Lehrkräfte in ihrem Unterricht im besten Fall an den individuellen Bedürfnissen ihrer Schüler*innen orientieren, ist anzunehmen, dass auch die elterliche Lernbegleitung adaptiv erfolgt. Daher nehmen wir sowohl die individuellen Ressourcen des Kindes als auch die wahrgenommene schulische Lernbegleitung als weitere Prädiktoren in den Blick.

### Merkmale des Elternhauses

Damit Eltern überhaupt das Lernen begleiten können, sind hinreichende zeitliche Ressourcen notwendig. Die zeitlichen Möglichkeiten werden unter anderem von elterlicher Erwerbstätigkeit sowie von der Gelegenheit, anfallende familiäre Aufgaben – auch jenseits der Lernunterstützung – auf mehrere Personen verteilen zu können beeinflusst. Gerade Alleinerziehende sind aufgrund der Vielzahl an familien- und berufsbezogenen Aufgaben besonders in ihrem Zeitbudget eingeschränkt. Wild und Gerber (2007) zeigen, dass Schüler*innen in der 7. Jahrgangsstufe mit vollberufstätigen Müttern im Vergleich zu teilzeitbeschäftigen bzw. erwerbsarbeitslosen Müttern überdurchschnittlich häufig keine elterliche Unterstützung bei den Hausaufgaben erhielten (42 % vs. 14 % bzw. 25 %). Fritzler und Wild (2019) weisen in einer Studie zur elterlichen Unterstützung von Kindern mit Lese-Rechtschreibstörung darauf hin, dass berufstätige Eltern grundsätzlich wenig Zeit haben, sich um die Bildungsprozesse der Kinder zu kümmern, und daher vermehrt auf externe Unterstützungsstrukturen zurückgreifen. Es kann daher angenommen werden, dass der Umfang der erwerbsbezogenen Zeitressourcenbindung im Zusammenhang mit den Möglichkeiten der elterlichen Lernunterstützung steht.

Hinsichtlich des sozioökonomischen Status (SES) wird vielfach darauf hingewiesen, dass Kinder aus Familien, die über einen höheren SES verfügen, mehr Unterstützung im Lernen erfahren und deutlich breiter in ihrer außerschulischen Bildung gefördert werden (z. B. Ditton und Maaz 2015). Neben der fachlichen Lernbegleitung können zudem ressourcenbezogene Faktoren des Haushalts einen Einfluss auf die Lernbegleitung und die Leistung der Schüler*innen haben. Hierzu zählen häusliche Ausstattungsaspekte wie ein Schreibtisch, ein ruhiger Platz zum Arbeiten, ein Computer, technische Nachschlagewerke oder ein Wörterbuch (vgl. OECD 2010).

Für Zusammenhänge mit der in der Familie gesprochenen Sprache liegt ein deutlich uneinheitliches Befundmuster vor. Luplow und Smidt (2019) berichten beispielsweise in einer Studie zur elterlichen Unterstützung von Grundschulkindern, dass Familien, in denen (in etwa) gleichhäufig Deutsch *und* eine andere Sprache gesprochen wird, weniger elterliche Unterstützung leisten als Familien, die überwiegend Deutsch oder überwiegend eine andere Sprache sprechen. Die fehlende Unterstützung scheint daher nicht auf sprachliche Problemlagen zurückführbar zu sein. Segeritz et al. (2010) untersuchten auf Basis von Daten der PISA-Studie 2003 die elterliche Hausaufgabenunterstützung in Abhängigkeit vom Geschlecht des Kindes und des Herkunftslandes. Insgesamt erhielten Kinder ohne Migrationshintergrund häufiger Hausaufgabenunterstützung als Kinder aus der ehemaligen UdSSR, aus Polen oder aus der Türkei. Mädchen wurden häufiger unterstützt als Jungen, wobei der Geschlechtsunterschied nur in der Personengruppe mit türkischer Herkunftsgeschichte statistisch signifikant ausfällt.

Inkonsistente Befunde lassen sich auch für das Bildungsniveau der Eltern zeigen. Zum einen berichten nationale wie internationale Studien, dass kein Zusammenhang zwischen der Quantität elterlicher Unterstützung und dem formalen Bildungsabschluss der Eltern besteht (Lee und Bowen 2006; Wild und Gerber 2007). Zum anderen weisen Studien darauf hin, dass mit einem höheren elterlichen Bildungsniveau eine höhere Unterstützungskompetenz einhergeht (z. B. Dumont et al. 2012; Niggli et al. 2007) und die Hausaufgabenunterstützung durch Eltern mit einem niedrigeren Bildungsabschluss negativ mit der schulischen Leistung des Kindes korreliert (vgl. Rønning 2011).

### Merkmale des Kindes

Die Schulschließung, aber auch der häusliche Distanzunterricht implizierten den Wegfall von zeitlichen Strukturen (z. B. Stundenplan, Unterrichtsphasen) und erschwerten die Möglichkeit Lernstrategien und -ziele vorzugeben. Gute schulische Leistungen sowie ausgeprägte selbstregulative Fähigkeiten des Kindes bzw. die Anwendung adäquater Lernstrategien können diesen Mangel vermutlich in Teilen kompensieren (Fischer et al. 2020). Ist auch dies nicht gegeben, erscheint es plausibel anzunehmen, dass die Eltern die strukturierende und fachlich unterstützende Funktion der Lehrkraft übernehmen. Genauere Annahmen dazu werden im Folgenden aus der Forschung zum elterlichen Involvement und insbesondere zur Hausaufgabenhilfe abgeleitet.

Entsprechend der obigen Erwartung konnte gezeigt werden, dass Eltern von Kindern, die im Unterricht gut mitkommen und gute Schulnoten haben, weniger fachliche Lernunterstützung leisten als Eltern von Kindern mit schwachen Leistungen (Helmke et al. 2004; Wild und Gerber 2007). In einer Studie von Dumont et al. (2014) unterstützten Eltern ihre Kinder zwar sowohl bei guten als auch bei schlechten Schulleistungen bei den Hausaufgaben, die Unterstützung wurde von den Kindern allerdings unterschiedlich wahrgenommen. Fünftklässler*innen mit schwachen Schulleistungen erlebten im siebten Schuljahr vermehrt elterliche Kontrolle. Kinder mit guten Leistungen in Klasse fünf berichteten zwei Jahre später zwar auch von einer hohen Lernunterstützung durch ihre Eltern, diese fand allerdings eher punktuell und auf gezielte Nachfrage statt. Gemäß den Überlegungen von Hoover-Dempsey et al. (2005) werden schwache Leistungen von den Eltern als Aufforderung oder „Einladung“ verstanden, das Kind auch ohne Nachfrage verstärkt zu unterstützen. Grolnick (2003) formuliert entsprechend, dass geringe Leistungen als „pressure from below“ wahrgenommen werden, die verstärkt in kontrollierendem Verhalten resultieren.

In gleicher Weise ist bei fehlenden selbstregulativen Fähigkeiten seitens der Kinder anzunehmen, dass Eltern strukturierend in das Lernen der Kinder eingreifen. Da die Vermittlung respektive Anwendung entsprechenden Strategiewissens an Gymnasien höher ausfällt als an nicht-gymnasialen Schulformen (Artelt et al. 2009; Dumke und Wolff-Kollmar 1997), ist mit weniger elterlicher Strukturierungshilfe zu rechnen, wenn das Kind ein Gymnasium besucht. Empirische Studien bestätigen diese Annahme, nach der Schülerinnen und Schüler am Gymnasium seltener von ihren Eltern beim häuslichen Lernen unterstützt werden als Kinder an nicht-gymnasialen Schulformen (Helmke et al. 2004; Wild und Gerber 2007). Gleichzeitig ist jedoch zu berücksichtigen, dass die Art der Hilfestellung für Kinder an Gymnasien eher funktional bzw. bedarfsorientiert erfolgt (Wild und Gerber 2007).

Mit Blick auf das Geschlecht ist die Befundlage weniger eindeutig. Einzelne Studien weisen darauf hin, dass Mädchen höhere selbstregulative Fähigkeiten (Duckworth und Seligman 2006) sowie stärker ausgebildete Arbeitstugenden (Anders et al. 2010) zugesprochen werden. Eine adaptive Lernbegleitung würde entsprechend eine geringere Strukturierung des Lernens durch die Eltern implizieren. Hingegen weisen Pomerantz und Ruble (1998) darauf hin, dass im Jugendalter von Jungen eine höhere Autonomie und Selbstständigkeit erwartet wird als von Mädchen. Aktuell ist uns nur eine Studie bekannt, die die Hausaufgabenunterstützung an weiterführenden Schulen mit dem Geschlecht der Schüler*innen in Beziehung setzt. In dieser Studie werden an weiterführenden Schulen – nicht aber an Grundschulen – die Mädchen von ihren Eltern stärker unterstützt als ihre männlichen Mitschüler (Cooper et al. 2000).

Neben Leistungen und Fähigkeiten werden möglicherweise auch weitere, motivational relevante Bedürfnisse des Kindes von den Eltern berücksichtigt. Gemäß der stage-environment-fit Theorie (Eccles et al. 1993) steigt das Bedürfnis nach Autonomie im Jugendalter an. In Übereinstimmung dazu zeigt die Forschung zur Hausaufgabenunterstützung eindrücklich, dass gerade im Jugendalter strikt kontrollierende Verhaltensweisen der Eltern negative Konsequenzen für die Lernentwicklung haben (Dumont et al. 2014). Dass Eltern auf dieses wachsende Autonomiebedürfnis angemessen reagieren können, zeigt die Untersuchung von Cooper et al. (2000) mit rund 700 Eltern von Schüler*innen aus den Klassenstufen zwei bis zwölf. Mit zunehmender Klassenstufe berichteten die Eltern, dass sie ihren Kindern mehr Freiheiten zustanden, sich weniger um die Rahmenbedingungen kümmerten, unter denen die Hausaufgaben bearbeitet wurden, und seltener fachliche Hilfe leisteten. Auch Wild und Gerber (2007) berichten auf Basis einer Längsschnittstudie mit gut 100 Eltern von Klasse fünf bis sieben von einem deutlichen Anstieg des Anteils derjenigen, die ihr Kind grundsätzlich alleine arbeiten lassen (von 67,3 % auf 82,5 %).

### Merkmale der (wahrgenommenen) schulischen Lernbegleitung

Bei der Abwägung, wie stark und in welcher Form sich Eltern bei der Begleitung des Lernens ihrer Kinder engagieren, werden – so unsere Annahme – neben Merkmalen des Kindes auch die schulischen Anforderungen bzw. Unterstützungsangebote eine Rolle spielen. So erscheint es zunächst plausibel anzunehmen, dass Eltern „kompensatorisch“ reagieren, indem sie sich insbesondere dann einbringen, wenn sich ihr Kind von den schulischen Aufgaben überfordert fühlt oder wenn sie die Lehrkraft als wenig engagiert wahrnehmen.

Aus der Forschung zu Bildungspartnerschaften (home-school-partnership oder family-teacher-partnership) ist jedoch bekannt, dass eine positive Einschätzung der Lehrkräfte durch die Eltern und eine gute Kommunikation wichtige Prädiktoren für elterliches Engagement sind (Denessen et al. 2007; Leenders et al. 2019; Tett und Macleod 2020). So illustrieren McNamara et al. (2000) in ihrer qualitativen Studie, dass das elterliche Engagement durch Lehrkraftäußerungen, die von den Eltern entweder als abwertend oder als überfordernd erlebt werden, gebremst wird, während wertschätzende Kommunikation und explizite Hinweise, wie Eltern dem Kind helfen können, das Engagement fördern. Diese Befunde lassen also im Gegensatz zu der „Kompensationsannahme“ erwarten, dass Eltern sich gerade dann stärker um das Lernen ihrer Kinder kümmern, wenn sie das Verhalten der Lehrkraft als engagiert und positiv erleben.

Im Rahmen dieser Studien wird immer wieder betont, dass das Involvement bei Familien mit Migrationshintergrund oder niedrigem sozioökonomischem Status eher gering ausfällt. Als Erklärung für die geringere Beteiligung wird dabei auch auf mangelndes Wissen über Curriculum sowie schulische Strukturen und Prozesse hingewiesen (Denessen et al. 2007; Leenders et al. 2019; Lopez et al. 2001). Obwohl die Eltern sich beteiligen wollen, fehlen ihnen kulturelle Ressourcen und das erforderliche Wissen, wie eine angemessene Beteiligung gelingen kann. Gut gestaltetes Arbeitsmaterial, klare Anweisungen und Rückmeldungen stellen somit möglicherweise für Eltern aus bildungsfernen Elternhäusern wichtige Hilfsmittel bei der Unterstützung ihrer Kinder dar. Vorstellbar ist auch, dass das Verhalten der Lehrkräfte eine Art Modell für diejenigen Eltern darstellt, die weniger gut über schulische Prozesse informiert sind. Positives Lehrkrafthandeln könnte für sie als Vorbild dienen, sich auch selbst besonders einzubringen.

## Forschungsfragen und Hypothesen

Auf Basis der Forschungsbefunde zur elterlichen Hausaufgabenunterstützung und zu Schule-Elternhaus-Partnerschaften leiten wir folgende Annahmen über die elterliche Lernbegleitung von Kindern und Jugendlichen an weiterführenden Schulen während der Phase der durch die Covid 19-Pandemie bedingten Schulschließung ab:

Lernbegleitung unterscheidet sich in Abhängigkeit von *soziodemographischen Merkmalen der Elternschaft*. Insbesondere wird – aufgrund geringerer zeitlicher Ressourcen – weniger Lernbegleitung erwartet, wenn die Eltern in hohem Umfang erwerbstätig oder alleinerziehend sind. Gleichzeitig sollte eine geringere schulische Vorbildung – aufgrund geringerer Fachkenntnisse – weniger prozessbezogene Lernbegleitung implizieren. Ein Effekt auf die strukturierende Begleitung wird hingegen nicht erwartet.

Unterschiede werden weiterhin in Abhängigkeit von *Merkmalen des Kindes* (Geschlecht, Leistungsstärke, besuchte Schulform) erwartet. Dabei gehen wir davon aus, dass Eltern ihre Unterstützung an die Leistungen und Potenziale des Kindes anpassen. In welche Richtung Unterschiede in Abhängigkeit vom Geschlecht des Kindes weisen, kann auf Basis der bisherigen Befundlage nicht vorhergesagt werden.

Ebenfalls erwarten wir einen Einfluss der von den Eltern *wahrgenommenen schulischen Lernbegleitung* auf das eigene Unterstützungshandeln, insbesondere mit Blick auf die prozessbezogene Lernbegleitung. Die Vorgabe einer Richtung der Effekte erweist sich als schwierig, da sowohl ein kompensatorisches als auch ein das Lehrkraftverhalten verstärkendes/nachahmendes Elternverhalten plausibel erscheint. Davon ausgehend, dass bildungsferne Eltern weniger klare Vorstellungen von Lern- und Lehrprozessen haben und daher stärker auf Hinweise und Anleitung der Lehrkraft angewiesen sein könnten, vermuten wir schließlich eine *Interaktion* zwischen dem elterlichen Schulabschluss und der Qualität des Lehrkrafthandelns derart, dass das Lehrkrafthandeln in bildungsfernen Familien stärker auf das elterliche Unterstützungshandeln wirkt als in bildungsnahe Familien.

## Methoden

### Design

Das Projekt „Familie und Schule in Zeiten der Corona-Pandemie (FamiSchul)“ untersucht die elterliche Wahrnehmung der Betreuung und Lernbegleitung während der Schulschließungen in Nordrhein-Westfalen vor den Osterferien 2020 aufgrund der Corona-Pandemie. Hierzu wurde eine querschnittliche Onlinebefragung konzipiert, die vom 15. April bis zum 11. Mai 2020 für Teilnehmende verfügbar war. Die Bearbeitungszeit lag bei *Med* = 20,17 min (*M* = 23,42 min). Es wurden sechs inhaltliche Frageblöcke zu folgenden Themenbereichen angeboten: (1) Zur familiären Situation, (2) Informationen zum Kind, (3) zur Lernbegleitung, Lernressourcen und lernunterstützenden Ressourcen, (4) Fragen zu Lernmaterialien und Einschätzungen von Schule und Lehrkräften, (5) soziodemographische Informationen der teilnehmenden Person und Sorgeneinschätzungen sowie (6) Einschätzungen der Vor- und Nachteile der Schulschließungen. Der Befragungsaufwand für die Eltern wurde dadurch reduziert, dass die schul-, lehrkraft- und lernbezogenen Informationen nur für das jeweils jüngste Kind an einer weiterführenden Schule erfragt wurden.

Für die Studiendissemination wurden verschiedene Wege verwendet: Die Landeselternschaften^1^ der Gymnasien, der integrierten Schulen und der Realschulen in NRW wurden kontaktiert und um eine Verbreitung über die ihnen zur Verfügung stehenden Verteiler gebeten. Zusätzlich wurden die Schulleitungen von 300 weiterführenden Schulen kontaktiert, auf persönliche Netzwerke zurückgegriffen sowie Informationstexte auf den Homepages der Arbeitsgruppe Forschungsmethoden/Empirische Bildungsforschung, des Instituts für Erziehungswissenschaft sowie der Westfälischen Wilhelms-Universität als weitere Verbreitungsvarianten eingesetzt.

### Stichprobe

Insgesamt absolvierten 6685 Personen den Fragebogen vollständig, das heißt, dass alle Befragungsblöcke des Onlinefragebogens angeschaut und somit die Befragung bis zum Ende absolviert wurde. 57 Personen wurden ausgeschlossen, da sie trotz „Vollständigkeit“ eine hohe Anzahl von mindestens 50 % fehlenden Werten (in den numerischen Variablen) aufwiesen. Somit liegt das Analysesample bei *N* = 6628 Personen.

Die befragten Personen sind zum Großteil weiblich (85,5 %) und zwischen 40 und 49 Jahre alt (59 %). Der häufigste Schulabschluss ist die Allgemeine Hochschulreife (54,8 %), der größte Anteil des höchsten beruflichen Abschlusses ist der Hochschulabschluss (39,7 %; vgl. Tab. 1 für eine Übersicht der Abschlüsse und einen Vergleich mit Daten der amtlichen Statistik für NRW).

**Table 1:** 

Höchster Schulabschluss			Höchster beruflicher Abschluss		
	Stichprobe	Amtliche Statistik NRW 2019 (40 bis 50-jährige)^a^		Stichprobe	Amtliche Statistik NRW 2019 (40 bis 50-jährige)^b^
Allgemeine Hochschulreife	54,8 %	42,34 %	Promotion	4,4 %	21,34 %
Fachhochschulreife	18,0 %		Hochschulabschluss	39,7 %	
Mittlere Reife	22,7 %	28,20 %	Techniker‑/Meisterschule	4,4 %	8,65^c^
Hauptschulabschluss, Klasse 10	2,8 %	22,53 %	Fachschule	13,4 %	
Hauptschulabschluss	1,0 %		Lehre	30,9 %	48,35 %^d^
Sonstige	0,7 %	–	Keinen beruflichen Abschluss	3,3 %	21,65 %^e^
Keinen allgemeinen Schulabschluss	–	6,85 %	Sonstige	3,9 %	–

^a^Eigene Berechnungen auf Basis von Daten, die vom Landesbetrieb IT.NRW Statistik und IT-Dienstleistungen (IT.NRW) auf Basis des Mikrozensus öffentlich zur Verfügung gestellt werden: Tabelle Bevölkerung in Privathaushalten ab 15 Jahren 2019 nach höchstem allgemeinen Schulabschluss, Geschlecht und Altersgruppen (https://www.it.nrw/statistik/eckdaten/bevoelkerung-privathaushalten-ab-15-jahren-nach-hoechstem-allgemeinen; Stand: 14.07.2020)

^b^Eigene Berechnung auf Basis von Daten, die von IT.NRW auf Basis des Mikrozensus öffentlich zur Verfügung gestellt werden: Tabelle Bevölkerung in Privathaushalten ab 15 Jahren 2019 nach höchstem beruflichen Ausbildungsabschluss, Geschlecht und Altersgruppe (https://www.it.nrw/statistik/eckdaten/bevoelkerung-privathaushalten-ab-15-jahren-nach-hoechstem-beruflichen; Stand: 14.07.2020)

^c^Fachschullabschluss und Fachschullabschluss in der ehemaligen DDR

^d^Lehre/Berufsausbildung im dualen System

^e^Hierunter fallen jedoch auch diejenigen Personen ohne Angabe sowie mit Anlernausbildung, beruflichem Praktikum oder Berufsvorbereitungsjahr. Letzteres wird jedoch in dieser Altersgruppe eher keine bedeutenden Ausmaße annehmen

Der Großteil der befragten Personen gibt an in der Familie (fast) nur Deutsch zu sprechen (87,9 %). Die durchschnittliche Haushaltsgröße liegt bei ca. vier Personen (*M* = 3,94; *SD* = 1,01), davon zwei Kinder (*M* = 2,39; *SD* = 1,36). Die Wohnlage der befragten Personen ist primär urban: 16,3 % Großstadt, 26,2 % Randgebiet oder Vorort einer Großstadt, 37,1 % Mittel- oder Kleinstadt, 20,4 % ländliches Dorf. Die Wohnung oder das Haus der befragten Personen verfügt durchschnittlich über ca. fünf Räume, wobei das Verhältnis von Räumen zu Personen bei ca. *M* = 1,35 (*SD* = 0,44) liegt. 95,1 % der befragten Personen gab an, dass ihre Familie über einen Garten oder eine fußläufig erreichbare Grünfläche verfügt. Als Indikator für das sozioökonomisch-kulturelle Kapital wurden die Bücher im Haushalt erhoben (vgl. Schwippert 2019 für eine Diskussion der Robustheit dieses Indikators): 10,4 % der Haushalte sind mit maximal 25 Büchern ausgestattet, 28,4 % mit 26–100 Büchern, 20,6 % mit 101–200 Büchern und 40,5 % mit über 200 Büchern.

Da nur nach dem jeweils jüngsten Kind der befragten Personen an einer weiterführenden Schule gefragt wurde, ist es nicht überraschend, dass ca. 65 % der Kinder die 5. bis 7. Klassenstufe besuchen. Das Alter der Kinder in der Gesamtstichprobe liegt bei ca. 12,5 Jahren (*M* = 12,64; *SD* = 1,96). Der Großteil der Lernenden besucht ein Gymnasium (70,5 %), gefolgt von Gesamt- (19,5 %) und Realschule (5,8 %). Diese Verteilung entspricht nicht der Schulformverteilung von Lernenden in Nordrhein-Westfalen, da die Kinder am Gymnasium in unserer Stichprobe deutlich überrepräsentiert sind.

Unter Berücksichtigung der Entstehung der Stichprobe und der dargestellten Stichprobencharakteristika ist nicht von einer repräsentativen Stichprobe zu sprechen. Die Betrachtung von erhobenen bildungsbezogenen Merkmalen und sozioökonomisch-kulturellen Ressourcen zeigt, dass Personen mit günstigeren Ausprägungen in diesen Bereichen in dieser Stichprobe deutlich überrepräsentiert sind.

### Instrumente und Operationalisierung

#### Lernbegleitung (angelehnt Quellenberg 2009):

Die *strukturierende Lernbegleitung *(*M* = 3,47; *SD* = 0,59) wurde mit drei Items erfasst. Beispielitem: „Ich/wir haben darauf geachtet, dass das Kind regelmäßige Lern‑/Arbeitszeiten nutzt“. Die *prozessbezogene Lernbegleitung* (*M* = 2,96; *SD* = 0,84) wurde ebenfalls mit drei Items erfasst. Beispielitem: „Ich/wir haben dabei geholfen sinnvolle Lernmethoden anzuwenden“. Die Antwortoptionen lagen auf einer vierstufigen Zustimmungsskala von 1 = „stimmt gar nicht“ bis 4 = „stimmt genau“. Die interne Konsistenz kann als zufriedenstellend bis gut interpretiert werden (α = 0,76 bzw. α = 0,87).

#### Teilnehmende

*Geschlecht: *Das Geschlecht wurde mit den drei Antwortoptionen *weiblich, männlich, divers* erhoben und für die Analysen dichotomisiert: *Frau* (ja/nein).

*Alter: *Die fünf Alterskategorien <30, 30–39, 40–49, 50–59 und >60 Jahre wurden zu drei Kategorien zusammengefasst und Dummy-kodiert: ≤39 Jahre, 40–49 Jahre und ≥50 Jahre. Die mittlere und anteilig größte Gruppe wurde als Referenzkategorie verwendet.

*Gesprochene Sprache: *Die in der Familie gesprochene Sprache wurde über ein dreistufiges Antwortformat erfragt: (fast) nur Deutsch, Deutsch und eine andere Sprache, (fast) nur eine andere Sprache. Die letzten beiden Antwortmöglichkeiten wurden für die Analysen zusammengefasst und in eine Variable *Familiensprache: (auch) nicht-deutsch* (12 %) rekodiert. Es ist einerseits zu betonen, dass dieser Indikator sehr grob ist und andererseits darauf hinzuweisen, dass von einer Unterrepräsentation von Personen auszugehen ist, die geringe Deutschkenntnisse aufweisen, da die Sprache der Befragung ausschließlich Deutsch war.

*Alleinerziehend: *Die Frage nach dem Zusammenleben mit einem Partner/einer Partnerin wurde bei Verneinung als Indikator für die Situation als alleinerziehende Person (14,5 %) verwendet.

*Sozioökonomisches-kulturelles Kapital*: Als Indikator für das sozioökonomisch-kulturelle Kapital wurden die Elternteile gefragt „Wie viel Bücher gibt es in Ihrem Haushalt ungefähr?“ (vgl. Wendt et al. 2016). Es konnte in fünf Kategorien geantwortet werden: 0–10, 11–25, 26–100, 101–200 und über 200. Für die Analysen wurde der heimische Buchbesitz in vier Dummy-Variablen kodiert und dabei die unteren beiden Kategorien zusammengefasst. Die Kategorie *über 200 Bücher *dient als Referenzkategorie.

*Elterlicher Schulabschluss*: Für den jeweiligen *höchsten Schulabschluss* der befragten Person wurden folgende Kategorien vorgegeben (angelehnt an Hoffmeyer-Zlotnik et al. 2010): (Allgemeine und Fach‑)Hochschulreife, Mittlere Reife, Hauptschulabschluss (Kl. 9 und Kl. 10), keinen allgemeinen Schulabschluss, sonstige Abschlüsse. Für die primären Analysen wurde der höchste Schulabschluss dichotomisiert in (Fach‑)Hochschulreife (72,8 %) bzw. keine (Fach‑)Hochschulreife (27,2 %).

*Arbeitszeit: *Die Arbeitszeit der Teilnehmenden (*M* = 25,56; *SD* = 14,60) wurde über die Frage erfasst: „Wie viele Wochenstunden beträgt Ihre tatsächliche Arbeitszeit derzeit?“. Es konnte eine Dezimalzahl mit zwei Nachkommastellen eingetragen werden. Die in den Analysen verwendete Variable wurde aufgrund von fehlenden Werten erweitert und durch Informationen über den/die Partner/in und unter Berücksichtigung der Erwerbssituation ergänzt, z. B. bei Vorhandensein eines Partners/einer Partnerin und jeweiliger Erwerbslosigkeit wurde die Arbeitszeit auf 0 gesetzt.

#### Merkmale des Kinders

*Alter: *Das Alter des jüngsten Kindes (*M* = 12,64; *SD* = 1,96) an einer weiterführenden Schule wurde metrisch erfasst.

*Geschlecht*: Das Geschlecht wurde ebenfalls dreistufig erfragt und für die Analysen dichotomisiert: *Mädchen* (ja: 48,9 %/nein: 51,1 %).

*Lern- und Arbeitsverhalten: *Das Lern- und Arbeitsverhalten des Kindes (*M* = 2,70; *SD* = 0,66) wurde über sieben Items eingeschätzt (angelehnt an Wendt et al. 2016). Beispielitem: „Mein Kind braucht für schulische Aufgaben wenig Hilfe“. Die Antwortoptionen lagen auf einer vierstufigen Zustimmungsskala von 1 = „stimmt gar nicht“ bis 4 = „stimmt genau“. Die interne Konsistenz kann als gut bezeichnet werden (α = 0,91).

*Schulform*: Zur Erfassung der Schulform des jeweils jüngsten Kindes auf einer weiterführenden Schule wurden die in NRW vorhandenen regulären Schulformen verwendet: Primusschule, Gemeinschaftsschule, Hauptschule, Realschule, Sekundarschule, Gesamtschule, Gymnasium. Die Schulform geht als dichotome Variable *Gymnasium *(ja: 70,5 %/nein: 29,5 %) in die Analysen ein.

#### Schulbezogene Variablen

*Engagement:* Das von den Eltern wahrgenommene Engagement der Lehrkräfte (*M* = 2,34; *SD* = 0,70) in Bezug auf Aufgaben und Lernunterstützung wurde über vier Items erfasst. Beispielitem: „Die meisten Lehrkräfte haben Aufgabenlösungen korrigiert und dann kommentiert zurückgeschickt“. Die schon zuvor dargestellte vierstufige Zustimmungsskala wurde eingesetzt. Die interne Konsistenz kann als zufriedenstellend bezeichnet werden (α = 0,77).

*Leistungsanforderungen: *Die elterliche Einschätzung der Leistungsanforderungen (*M* = 2,22; *SD* = 0,71) wurde über drei Items erfasst. Beispielitem: „Die Leistungsanforderungen an mein Kind waren im Rahmen der aktuellen besonderen Situation zu hoch“. Die schon zuvor dargestellte vierstufige Zustimmungsskala wurde verwendet. Die interne Konsistenz kann als zufriedenstellend bezeichnet werden (α = 0,77).

### Auswertungsstrategie

Es wurden schrittweise lineare Regressionsmodelle verwendet. Für das erste Modell wurden elterliche/familiäre soziodemographische Variablen einbezogen. In einem zweiten Modell wurden die Merkmale der Kinder und die Einschätzung zur Schule sowie Interaktionsterme zwischen elterlichem Bildungshintergrund und den schulbezogenen Variablen hinzugefügt.

## Ergebnisse

### Deskriptiva

Die Korrelationen zwischen den in den Regressionsanalysen verwendeten Variablen finden sich in Tab. 2. Es werden nachfolgend nur moderate Korrelationen berichtet. Die beiden Kriterien der Regressionsanalysen *strukturierende Begleitung* und *prozessbezogene Begleitung* korrelieren moderat (*r* = 0,45, *p* ≤ 0,001). Die Prädiktoren korrelieren überwiegend schwach, wenngleich statistisch signifikant. Moderate positive Korrelationen finden sich jedoch zwischen elterlicher *(Fach‑)Hochschulreif*e und *Buchbesitz im Haushalt* (*r* = 0,40, *p* ≤ 0,001),* Alter des Kindes* und *Alter der Eltern* (*r* = 0,33, *p* ≤ 0,001) sowie zwischen *Gymnasialbesuch des Kindes* und elterlicher* (Fach‑)Hochschulreife* (*r* = 0,32, *p* ≤ 0,001). Gering eingeschätztes *Arbeits- und Lernverhalten* des Kindes gehen mit stärkerer Wahrnehmung einer zu hohen *Leistungsanforderung* durch die schulischen Aufgaben einher (*r* = −0,33, *p* ≤ 0,001).

**Table 2:** 

	*M* (SD)	(1)	(2)	(3)	(4)	(5)	(6)	(7)	(8)	(9)	(10)	(11)	(12)	(13)	(14)
(1) Strukturierende Lernbegleitung	3,466 (0,586)	1	–	–	–	–	–	–	–	–	–	–	–	–	–
(2) Prozessbezogene Lernbegleitung	2,959 (0,835)	0,450^***^	1	–	–	–	–	–	–	–	–	–	–	–	–
(3) Frau	85,5 %	0,042^***^	0,026^*^	1	–	–	–	–	–	–	–	–	–	–	–
(4) Alter	59,0 %^a^	−0,143^***^	−0,210^***^	−0,206^***^	1	–	–	–	–	–	–	–	–	–	–
(5) Fam.-sprache: (auch) nicht-deutsch	12,0 %	0,028^*^	0,070^***^	−0,049^***^	−0,119^***^	1	–	–	–	–	–	–	–	–	–
(6) Alleinerziehend	19,4 %	−0,033^*^	−0,007	0,013	0,033^*^	−0,025	1	–	–	–	–	–	–	–	–
(7) Bücher im Haushalt	40,6 %^b^	0,007	−0,115^***^	0,003	0,251^***^	−0,119^***^	−0,094^***^	1	–	–	–	–	–	–	–
(8) (Fach‑)Hochschulreife	72,8 %	−0,006	−0,096^***^	−0,076^***^	0,170^***^	−0,038^**^	−0,039^**^	0,403^***^	1	–	–	–	–	–	–
(9) Arbeitszeit	25,560 (14,595)	−0,068^***^	−0,069^***^	−0,324^***^	0,101^***^	−0,013	0,013	0,048^***^	0,099^***^	1	–	–	–	–	–
(10) Alter (Kind)	12,640 (1,959)	−0,292^***^	−0,401^***^	−0,026^*^	0,331^***^	−0,030^*^	0,059^***^	0,031^*^	−0,034^**^	0,072^***^	1	–	–	–	–
(11) Mädchen	48,86 %	−0,030^*^	−0,077^***^	0,012	0,015	0,023	0,016	0,003	−0,014	0,003	0,004	1	–	–	–
(12) Lern‑/Arbeitsverhalten	2,698 (0,660)	0,083^***^	−0,169^***^	−0,009	0,050^***^	0,044^***^	−0,033^*^	0,109^***^	0,075^***^	0,005	0,125^***^	0,250^***^	1	–	–
(13) Gymnasium	70,5 %	0,007	−0,158^***^	−0,038^***^	0,153^***^	−0,008	−0,056^***^	0,268^***^	0,319^***^	0,056^***^	−0,002	0,091^***^	0,230^***^	1	–
(14) Engagement	2,340 (0,704)	0,135^***^	0,094^***^	0,032^**^	−0,060^***^	0,051^***^	−0,016	−0,069^***^	−0,109^***^	−0,063^***^	−0,015	0,056^***^	0,278^***^	−0,017	1
(15) Leistungsanforderungen	2,217 (0,710)	−0,011	0,180^***^	0,017	−0,060^***^	0,021	0,021	−0,111^***^	−0,066^***^	−0,008	−0,110^***^	0,008	−0,334^***^	−0,081^***^	−0,264^***^

*Fam.-sprache* Familiensprache

^***^p ≤ 0,001; ^**^p ≤ 0,01; ^*^p ≤ 0,05

^a^Alterskategorie 40–49 Jahre

^b^Über 200 Bücher

### Vorhersage der strukturierenden Lernbegleitung

Die strukturierende Lernbegleitung hängt relativ schwach mit den soziodemographischen Merkmalen der befragten Eltern zusammen (vgl. Tab. 3). Es zeigt sich ein signifikanter Zusammenhang zwischen strukturierender Begleitung und dem Alter, wobei jüngere Eltern das Lernen stärker strukturierend begleiten als ältere (Referenzgruppe 40–49 Jahre; Alter <40: β = 0,04; *p* = 0,014; Alter ≥50: β = −0,13; *p* ≤ 0,001). Ebenfalls ist ein deutlicher negativer Zusammenhang mit der Berufstätigkeit nachweisbar: Je höher die Arbeitszeit der Befragten, desto weniger sorgen die Eltern für die Sicherung günstiger Rahmenbedingungen für das Lernen des Kindes (β = −0,07; *p* ≤ 0,001). Gleichzeitig geht ein geringes sozioökonomisch-kulturelles Kapital, erfasst über die Anzahl der Bücher, mit wenig strukturierender Begleitung einher. Dabei unterscheidet sich aber nur die Gruppe derjenigen mit weniger als 25 Büchern von der Referenzgruppe (Referenzgruppe >200 Bücher; 0–25 Bücher: β = −0,06; *p* ≤ 0,001). In Familien, die (auch) eine nicht deutsche Familiensprache sprechen, ist die strukturierende Begleitung stärker ausgeprägt als in ausschließlich deutschsprachigen Familien (β = 0,03; *p* = 0,024). Der Bildungshintergrund, operationalisiert über den Schulabschluss, und der Familienstatus sind bei Kontrolle der anderen Variablen nicht prädiktiv. Das Regressionsmodell, das nur die soziodemographischen Merkmale des Elternhauses als Prädiktoren berücksichtigt (Modell 1), erklärt nur 2,7 % der Varianz.

In Modell 2 wurden die Merkmale des Kindes und der schulischen Begleitung ergänzt. Unter Kontrolle dieser Merkmale bleiben die Alterskategorie ≥50 Jahre im Vergleich zu den 40–49-jährigen Teilnehmenden (β = −0,04; *p* = 0,025), die Gruppe mit den wenigen Büchern im Haushalt (0–25) im Vergleich mit denjenigen mit über 200 Büchern (β = −0,05; *p* = 0,003) sowie die Arbeitszeit als negative Prädiktoren (β = −0,04; *p* = 0,008) erhalten. Zusätzlich erweist sich die (Fach‑)Hochschulreife als signifikanter Prädiktor (β = −0,20; *p* = 0,016). Eltern mit höherem Bildungsabschluss sorgen sich weniger um die Sicherung der Arbeitsbedingungen für ihre Kinder.

Die Hinzunahme der weiteren Merkmale ergibt folgende Befunde: Die strukturierende Begleitung fällt umso geringer aus, je älter das Kind ist (β = −0,29; *p* ≤ 0,001). Mädchen erhalten weniger Unterstützung als Jungen (β = −0,07; *p* ≤ 0,001). Je positiver das Lern- und Arbeitsverhalten des Kindes eingeschätzt wird, desto stärker sorgen Eltern für gute Rahmenbedingungen (β = 0,12; *p* ≤ 0,001). Weiterhin kümmern sich Eltern, die das Engagement der Lehrkräfte positiv wahrnehmen, stärker um die strukturierende Begleitung des Lernens ihrer Kinder (β = 0,09; *p* ≤ 0,001). Eine Interaktion mit dem elterlichen Bildungsabschluss liegt nicht vor. Anders sieht es bei den Leistungsanforderungen aus. Diese sind zwar nicht prädiktiv für die strukturierende Begleitung (β = 0,05; *p* = 0,089), aber es liegt eine signifikante Interaktion mit dem elterlichen Bildungsabschluss vor (β = 0,19, *p* ≤ 0,001). Während also Eltern mit (Fach‑)Hochschulreife die strukturierende Begleitung unabhängig von den wahrgenommenen schulischen Anforderungen leisten, orientieren sich Eltern ohne (Fach‑)Hochschulreife an diesen Anforderungen. Sie berichten von verstärkter strukturierender Unterstützung, wenn sie die Anforderungen für ihr Kind als hoch einstufen. Das Modell erklärt insgesamt 12,1 % der Varianz der strukturierenden Lernbegleitung in den Familien.

**Table 3:** 

Strukturierende Lernbegleitung	Modell 1: Soziodemographie		Modell 2: Gesamt	
	β	SE	β	SE
**Demographische Merkmale**				
*Frau*	−0,006	0,026	0,012	0,025
*Alter (Ref.: 40–49 Jahre)*				
<40 Jahre	0,040^*^	0,027	0,003	0,026
≥50 Jahre	−0,126^***^	0,021	−0,036	0,021
*Familiensprache: (auch) nicht-deutsch*	−0,021	0,022	0,028^+^	0,027
*Alleinerziehend*	−0,021	0022	−0,007	0,021
*Bücher im Haushalt (Ref. >200 Bücher)*				
0–25	−0,059^***^	0,034	−0,051^**^	0,032
26–100	0,006	0,023	0,015	0,022
101–200	−0,015	0,024	−0,006	0,023
*(Fach‑)Hochschulreife (HSR)*	−0,004	0,022	−0,196^*^	0,108
*Arbeitszeit (in Std.)*	−0,068^***^	0,001	−0,041^*^	0,001
**Merkmale des Kindes**				
*Alter (in Jahren)*	–	–	−0,288^***^	0,005
*Mädchen*	–	–	−0,069^***^	0,017
*Lern‑/Arbeitsverhalten*	–	–	0,121^***^	0,015
*Gymnasialbesuch*	–	–	−0,007	0,020
**Schulische Lernbegleitung**				
*Engagement*	–	–	0,094^***^	0,024
*Leistungsanforderungen*	–	–	−0,052^+^	0,025
**Interaktionseffekte**				
HSR*Engagement	–	–	0,018	0,028
HSR*Leistungsanforderungen	–	–	0,187^***^	0,028
*R*^*2*^	–	0,027	–	0,121

^***^p ≤ 0,001; ^**^p ≤ 0,01; ^*^p ≤ 0,05; ^+^*p* ≤ 0,10

### Vorhersage der prozessbezogenen Begleitung

Die Vorhersage der prozessbezogenen Lernbegleitung durch die soziodemographischen Merkmale der Eltern ergibt folgende Befunde (vgl. Tab. 4, Modell 1): Es zeigt sich ein signifikanter Zusammenhang der prozessbezogenen Begleitung mit dem Alter, wobei jüngere Eltern den Lernprozess intensiver begleiten als ältere (Referenzgruppe 40–49 Jahre; Alter <40: β = 0,08; *p* ≤ 0,001; Alter ≥50: β = −0,14; *p* ≤ 0,001). Im Gegensatz zur strukturierenden Begleitung zeigt sich eine intensivere fachliche Begleitung des Lernprozesses bei Familien mit geringerem sozioökonomisch-kulturellen Kapital, erfasst über die Anzahl der Bücher (Referenzgruppe >200 Bücher; 0–25 Bücher: β = 0,05; *p* = 0,002; 26–100 Bücher: β = 0,04; *p* = 0,017). Eine (auch) nicht deutsche Familiensprache geht mit verstärkter Unterstützung des Lernprozesses einher (β = 0,04; *p* = 0,009). Ein deutlicher negativer Zusammenhang ist hingegen mit der Berufstätigkeit nachweisbar: Je höher die Arbeitszeit der Befragten, desto weniger kümmern sich die Eltern um den Lernprozess des Kindes (β = −0,05; *p* ≤ 0,001). Auch der Bildungshintergrund, operationalisiert über den Schulabschluss, ist negativ mit der prozessbezogenen Lernbegleitung assoziiert (β = −0,05; *p* = 0,004). Schließlich erweist sich der Familienstatus bei Kontrolle der anderen Variablen nicht als prädiktiv. Das Regressionsmodell, das nur die soziodemographischen Merkmale des Elternhauses als Prädiktoren berücksichtigt (Modell 1), erklärt 5,1 % der Varianz.

**Table 4:** 

Prozessbezogene Lernbegleitung	Modell 1: Soziodemographie		Modell 2: Gesamt	
	β	SE	β	SE
**Demographische Merkmale**				
*Frau*	−0,025	0,037	−0,009	0,033
*Alter (Ref.: 40–49 Jahre)*				
<40 Jahre	0,077^***^	0,038	0,015	0,035
≥50 Jahre	−0,141^***^	0,030	−0,024	0,028
*Familiensprache: (auch) nicht-deutsch*	0,040^**^	0,040	0,044^***^	0,36
*Alleinerziehend*	0,007	0,031	0,015	0,028
*Bücher im Haushalt (Ref. >200 Bücher)*				
0–25	0,054^**^	0,047	0,019	0,053
26–100	0,041^*^	0,032	0,020	0,029
101–200	0,006	0,034	0,001	0,031
*(Fach‑)Hochschulreife (HSR)*	−0,047^**^	0,031	−0,100	0,145
*Arbeitszeit (in Std.)*	−0,054^***^	0,001	−0,026^+^	0,001
**Merkmale des Kindes**				
*Alter (in Jahren)*	–	–	−0,354^***^	0,006
*Mädchen*	–	–	−0,053^***^	0,023
*Lern‑/Arbeitsverhalten*	–	–	−0,080^***^	0,020
*Gymnasialbesuch*	–	–	−0,094^***^	0,027
**Schulische Lernbegleitung**				
*Engagement*	–	–	0,187^***^	0,032
*Leistungsanforderungen*	–	–	0,058^*^	0,033
**Interaktionseffekte**				
HSR*Engagement	–	–	−0,102^+^	0,037
HSR*Leistungsanforderungen	–	–	0,177^***^	0,038
*R*^*2*^	–	0,051	–	0,229

^***^p ≤ 0,001; ^**^p ≤ 0,01; ^*^p ≤ 0,05; ^+^*p* ≤ 0,10

In nächsten Schritt (vgl. Tab. 4, Modell 2) wurden die Merkmale des Kindes und der schulischen Begleitung ergänzt. Unter Kontrolle dieser Merkmale verbleibt bei den soziodemographischen Variablen nur noch die Familiensprache als signifikanter positiver Prädiktor (β = 0,04; *p* ≤ 0,001). Jedoch sind alle vier kindbezogenen Merkmale signifikant. So fällt die Begleitung des Lernprozesses umso geringer aus, je älter das Kind ist (β = −0,35; *p* ≤ 0,001) und je positiver die Eltern dessen Lernverhalten einschätzen (β = −0,08; *p* ≤ 0,001). Weiterhin ist die Unterstützung geringer, wenn es sich um ein Mädchen handelt (β = −0,05; *p* ≤ 0,001) und wenn das Kind ein Gymnasium besucht (β = −0,09; *p* ≤ 0,001). Seitens der schulischen Variablen ist das wahrgenommene Engagement der Lehrkraft bedeutsam für die prozessbezogene Begleitung durch die Eltern (β = 0,19; *p* ≤ 0,001). Dieser Effekt wird jedoch durch eine (tendenziell) signifikante Interaktion qualifiziert (β = −0,10; *p* = 0,052). Dies bedeutet, dass sich die Eltern umso mehr um den Lernprozess des Kindes kümmern, je stärker das Engagement ist, das sie bei den Lehrkräften wahrnehmen. Dieser Zusammenhang fällt bei Eltern mit (Fach‑)Hochschulreife jedoch deutlich schwächer aus als bei Eltern mit niedrigerem Schulabschluss. Schließlich ist auch die wahrgenommene Leistungsanforderung mit der prozessbezogenen Lernbegleitung gekoppelt (β = 0,06; *p* = 0,044). Je höher die Anforderungen eingeschätzt werden, desto mehr wird in die Lernbegleitung investiert. Das signifikante Regressionsgewicht für die Interaktion (β = 0,18; *p* ≤ 0,001) besagt jedoch, dass dieser Zusammenhang bei Eltern mit (Fach‑)Hochschulreife noch deutlich stärker ausgeprägt ist als bei Eltern ohne (Fach‑)Hochschulreife. Das Modell klärt insgesamt 22,9 % der Varianz auf.

## Diskussion

Mit der vorliegenden Studie haben wir überprüft, inwiefern die familiäre Lernbegleitung einerseits durch soziodemographische Merkmale der Familie und andererseits durch kind- und schulbezogene Merkmale vorhergesagt werden kann. Dabei wurde zwischen der elterlichen Selbsteinschätzung der strukturierenden sowie der prozessbezogenen Begleitung des Lernprozesses unterschieden. Bevor die zentralen Befunde zusammenfassend eingeordnet, diskutiert und auf zukünftige Perspektiven sowie Praxisimplikationen eingegangen werden kann, werden nachfolgend zunächst methodische Limitationen der Studie thematisiert.

### Limitationen

Eine zentrale Limitation der vorliegenden Studie liegt in der selektiven Stichprobe, die aufgrund der gewählten Studiendissemination und der Onlinebefragung als Befragungsmethode resultierte. So ist anzunehmen, dass der hohe Anteil an Eltern mit Kindern, die ein Gymnasium besuchen, auch auf die starke Unterstützung bei der Weiterleitung der Befragung an interessierte Eltern durch die Landeselternschaft der Gymnasien zurückzuführen ist. Die parallele Kontaktierung der Landeselternschaften anderer Schulformen führte zu weniger Resonanz. Zusätzlich wurden daher 324 Schulleitungen von Real‑/Haupt‑/Gesamt- und Sekundarschulen angeschrieben und um Weiterleitung des Studienzugangs gebeten. Trotz dieser Versuche, eine gewisse Ausbalancierung der Stichprobe zu erreichen, ergibt sich – wie auch in anderen ad-hoc Studien – eine Stichprobenverzerrung, sodass Verallgemeinerungen auf die Grundgesamtheit aller Eltern in NRW nicht ohne weiteres möglich sind.

Die Problematik von nichtrepräsentativen Stichproben im Rahmen der Forschung auch zu bildungsbezogenen Auswirkungen liegt in vielen aktuellen Befragungen vor. Huber und Helm (2020) verweisen in ihrem Beitrag beispielsweise kurz auf die Problematik der ad-hoc Stichprobe ihrer Schulbarometererhebung von ca. 8300 Schüler*innen in Deutschland, Österreich und der Schweiz. Fickermann und Edelstein (2020) fordern im Editorial eines Themenheftes der Zeitschrift *Die Deutsche Schule *zu Recht eine Diskussion von Problemen aufgrund von Onlinebefragungen hinsichtlich der Repräsentativität. Auch fordern die beiden Autoren, dass die sozioökonomischen Merkmale berücksichtigt werden sollten. Dieser Forderung entsprechen beispielweise Porsch und Porsch (2020), die relativ ausführlich die ca. 4000 Eltern von Grundschulkindern umfassende Stichprobe beschreiben und abschließend auch die Repräsentativität der Erhebung kritisch einordnen. Wir kommen der Forderung von Fickermann und Edelstein nach und explizieren in der Stichprobenbeschreibung anhand der Schul- und Berufsabschlüsse die Selektivität der Stichprobe im Vergleich mit Daten der amtlichen Statistik für NRW.

Weitere Einschränkungen ergeben sich, da es sich um Daten aus einer Querschnittsbefragung handelt, die eine Kausalinterpretation nicht zulassen. So können beispielsweise Zusammenhänge zwischen der Wahrnehmung schulischer Unterstützung und der eigenen Lernbegleitung grundsätzlich in beide (Verursachungs‑)Richtungen interpretiert werden.

Schließlich ist darauf hinzuweisen, dass im Rahmen der thematisch breit angelegten Onlinebefragung die einzelnen Merkmale und Konstrukte nur mit wenigen Items abgefragt wurden. Eine differenziertere Erfassung der Kernvariablen wäre sicher wünschenswert gewesen, erschien aber mit Blick auf die Länge des Fragebogens nicht angeraten.

### Kernbefunde

Wir postulierten, dass sich alleinerziehende Eltern ebenso wie Personen mit hoher Stundenzahl für Erwerbstätigkeit aufgrund fehlender zeitlicher Ressourcen weniger um den Lernprozess ihrer Kinder kümmern können. Diese Annahme lässt sich für die Arbeitszeit, nicht aber für den Familienstatus bestätigen. Die Befunde aus früheren Studien zum Zusammenhang von Migrationshintergrund und elterlicher Unterstützung waren eher diffus (Luplow und Smidt 2019; Segeritz et al. 2010). In unserer Befragung zeigt sich eine leicht höhere Unterstützung für Kinder aus Familien mit nicht-deutscher Familiensprache, die eventuell auf ein kompensatorisches Verhalten der Eltern hinweist. Dieser Befund muss jedoch mit großer Vorsicht betrachtet werden, da vermutlich gerade die Stichprobe von Eltern mit nicht-deutscher Herkunftssprache besonders selektiv ist. Der umfangreiche Fragebogen wurde nur in deutscher Sprache angeboten, sodass Eltern, die Probleme mit der deutschen Sprache haben, sicher nicht teilgenommen haben.

Merkmale des sozioökonomisch-kulturellen Kapitals – operationalisiert über die Anzahl an Büchern im Haushalt und über den elterlichen Schulabschluss – erweisen sich nach Berücksichtigung von Merkmalen des Kindes allenfalls für die strukturierende Lernbegleitung als signifikante Prädiktoren. Eltern ohne (Fach‑)Hochschulabschluss bestätigen eher darauf zu achten, dass ihr Kind regelmäßig genug Zeit mit Lernen verbringt und die dafür erforderlichen Hilfsmittel zur Verfügung hat. Gleichzeitig unterscheiden sich aber auch Familien mit sehr wenigen Büchern (<25) und solche mit sehr vielen Büchern (>200) in der strukturierenden Lernbegleitung. Das Befundmuster ist zunächst schwierig zu deuten. Vertiefende Analysen unter Berücksichtigung weiterer familiärer Ausstattungsmerkmale könnten eventuell hilfreich sein. Es ist jedoch zu beachten, dass die Varianzaufklärung allein durch die soziodemographischen Variablen sehr gering ist, sodass diese Befunde nicht überinterpretiert werden sollten.

Insgesamt ist somit festzuhalten, dass die soziodemographischen Variablen Unterschiede in der Lernbegleitung nur in sehr geringer Weise erklären können. Wesentlich besser gelingt dies durch die Merkmale des Kindes und die wahrgenommene schulische Begleitung. Sowohl die strukturierende als auch die prozessbezogene Lernbegleitung der Eltern fällt höher aus, wenn das Kind ein Junge ist, ein eher ungünstiges Lern- und Arbeitsverhalten zeigt und jünger ist, also eine niedrige Klassenstufe besucht. Eltern helfen weniger bei der Bearbeitung der Aufgaben, wenn das Kind ein Gymnasium besucht. Die Sicherung günstiger Rahmenbedingungen durch eine strukturierende Lernbegleitung ist jedoch unabhängig von der Schulform. Unter Berücksichtigung der Annahmen, dass Mädchen stärker ausgebildete Arbeitstugenden zugesprochen werden (Anders et al. 2010; Krahé et al. 2007) und, dass am Gymnasium stärker als an anderen Schulformen selbstregulative Fähigkeiten ausgebildet werden (Artelt et al. 2009), lassen sich diese Befunde im Sinne eines adaptiven Unterstützungsverhaltens der Eltern interpretieren. Offen bleibt jedoch die Frage, ob die geleistete Unterstützung wirklich förderlich ist. Wiederholt wird in der Literatur der Befund genannt, dass schwache Leistungen des Kindes bei Eltern häufig ein dysfunktionales, bevormundendes Hilfeverhalten auslösen, das sich als motivational ungünstig erweist, sodass die damit letztlich angestrebte Leistungssteigerung ausbleibt (z. B. Dumont et al. 2014). Wenn in der Zeit der Schulschließung die Eltern über einen längeren Zeitraum die primäre Lernbegleitung des Kindes darstellen, steht zu befürchten, dass der negative Effekt besonders stark ausfallen könnte. Hier wäre es besonders wichtig, dass Schulen bzw. Lehrkräfte den Eltern Hinweise geben, wie sie ihre Kinder begleiten können, sodass ein lernförderlicher Effekt entsteht. Immerhin belegen die vorliegenden Daten durchaus, dass die Eltern bemüht sind, ihrem Kind zu helfen, gerade wenn sie Schwierigkeiten wahrnehmen oder antizipieren.

Interessante Zusammenhangsmuster zeigen sich auch mit Blick auf die wahrgenommene schulische Lernbegleitung. Insgesamt weisen die Daten auf einen positiven Zusammenhang des (wahrgenommenen) Engagements der Lehrkraft mit der elterlichen Lernbegleitung hin. Dieser Zusammenhang ist mit Blick auf die prozessbezogene Begleitung bei Eltern ohne (Fach‑)Hochschulabschluss besonders deutlich ausgeprägt. Hier liegt die Annahme nahe, dass Eltern das engagierte Verhalten der Lehrkraft als Modell für das eigene Handeln nutzen. Gleichzeitig kann angenommen werden, dass Lerntipps, Korrekturen und Kommentare, welche die Lehrkräfte an die Kinder richten, gerade für bildungsfernere Eltern hilfreiche Anregungen für die eigene Unterstützung ihrer Kinder liefern. In bildungsnahen Familien, in denen Eltern sowohl eine eigene klare Idee von Standards des Lernens und Möglichkeiten der Lernbegleitung haben, scheint das Verhalten der Lehrkraft weniger bedeutsam zu sein. Ebenfalls wird die elterliche Unterstützung des Lernprozesses verstärkt, wenn die gestellten Anforderungen als überfordernd für das Kind wahrgenommen werden. Ein derartiges adaptives Unterstützungsverhalten wird insbesondere in Familien mit hohem Schulabschluss der Eltern geleistet, während Familien mit niedrigerem Bildungsniveau ihre Unterstützung weniger stark an die Anforderungen anpassen.

Auch wenn die Analysen also keine direkten Unterschiede in der Lernbegleitung in Abhängigkeit von soziodemographischen Merkmalen des Elternhauses belegen, zeigen sich hier durch die Schule bedingte Effekte. So scheint die Qualität der schulischen Begleitung vor allem für Kinder aus Familien wichtig zu sein, in denen die schulische Bildung der Eltern geringer ausgeprägt ist. Dabei ist mit Blick auf die verzerrte Stichprobe damit zu rechnen, dass dieser Effekt in einer repräsentativen Stichprobe eventuell noch stärker zu Buche schlagen würde.

### Implikationen für die Praxis

Stellungnahmen und Expertisen weisen auf die besondere Gefahr der Ausweitung sozialer Bildungsungleichheiten hin, welche sich durch unterschiedliche familiäre Ausstattung mit notwendigen Ressourcen und individuellen Unterstützungsmöglichkeiten ergeben kann (z. B. van Ackeren et al. 2020). Die Ergebnisse der vorliegenden Studie liefern Hinweise darauf, dass ein engagiertes Lehrkraftverhalten während der Pandemie dazu beitragen kann, die elterliche Lernbegleitung zu aktivieren, insbesondere in Familien mit niedrigerem Bildungsniveau. Lehrkräfte können durch ihr Verhalten also vermutlich gleich in doppelter Hinsicht dazu beitragen, dass es im Kontext eines Distanzunterrichts nicht zu der befürchteten Verstärkung sozialer Ungleichheiten in der Lernentwicklung der Kinder kommt: Sie können für ihre Schüler*innen individualisiert Aufgaben auswählen (Moroni et al. 2014), gezielte Rückmeldung zu bearbeiteten Aufgaben geben und gegebenenfalls Hinweise zu adäquaten Lern- und Arbeitsstrategien ergänzen, sodass die Schüler*innen die Inhalte weitgehend selbstständig erarbeiten können (Voss und Wittwer 2020). Damit können sie gleichzeitig als „Modelle“ für die Eltern fungieren, die sich an der Rückmeldung und dem Engagement der Lehrkräfte vermutlich in ihrem eigenen Unterstützungshandeln orientieren.

Neben der Modellwirkung kann die direkte, an die Eltern gerichtete Kommunikation über konkrete elterliche Unterstützungsmöglichkeiten die positive Elternunterstützung fördern.

Bereits vor den Schulschließungen wurde in dem Bereich der elterlichen Hausaufgabenunterstützung die Forderung laut, Lehrkräfte bzw. die Schule sollten Eltern besser auf die Begleitung des Lernens ihrer Kinder vorbereiten (Dumont et al. 2014). Um den individuellen Bedürfnissen und Voraussetzungen der Eltern gerecht zu werden, könnten Lehrkräfte die Eltern über förderliche oder hemmende Formen der Lernbegleitung (Guill et al. 2019) informieren und im Bedarfsfall eine spezifische Beratung anbieten (Moroni et al. 2014). Studien belegen, dass die Qualität der elterlichen Hausaufgabenunterstützung über Trainings/Schulungen zur Einübung förderlicher Unterstützungsstrategien durchaus verbessert werden kann (Fantuzzo et al. 1995; Froiland 2011; Villiger et al. 2010). Dabei ist jedoch auch entscheidend, dass ein wertschätzender Kontakt und Austausch zwischen Eltern und Schule aufgebaut wird, da dieser zum elterlichen Engagement im Allgemeinen (McNamara et al. 2000; Tett und Macleod 2020; Wenzel 2020) und zur Wirkung eines Elterntrainings im Besonderen (Fantuzzo et al. 1995) beiträgt. Wenngleich die Etablierung von Elterntrainings oder der Aufbau positiver Bildungspartnerschaften zwischen Schule und Elternhaus kaum kurzfristig in der Krisensituation gelingen kann, legen die Daten der vorliegenden Studie es nahe, Lehrkräfte für die direkten und indirekten Effekte ihres Handelns zu sensibilisieren, sodass sie ihr eigenes Handeln diesbezüglich anpassen können. Es wäre zu wünschen, dass die vorliegenden Befunde weiterführende Untersuchungen zur aktivierenden Wirkung oder Modellfunktion engagierten Lehrkrafthandelns für Eltern – auch unter Berücksichtigung soziodemographischer Merkmale – anregen.

## Footnotes

1 Landeselternschaften sind ein Zusammenschluss von Eltern auf Bundeslandebene zur Mitwirkung im Bildungsprozess (rechtliche Grundlage: Schulgesetz für das Land Nordrhein-Westfalen § 62 Abs. 1).
